# Advances in the two-dimensional layer materials for cancer diagnosis and treatment: unique advantages beyond the microsphere

**DOI:** 10.3389/fbioe.2023.1278871

**Published:** 2023-09-29

**Authors:** Zheng-Wei Zhang, Yang Yang, Han Wu, Tong Zhang

**Affiliations:** ^1^ Department of Hepatopancreatobiliary Surgery, Xinghua People’s Hospital, Yangzhou University, Xinghua, Jiangsu, China; ^2^ Department of Hepatobiliary Surgery, Eastern Hepatobiliary Surgery Hospital, Naval Medical University, Shanghai, China; ^3^ Department of Hepatopancreatobiliary Surgery, The Third Affiliated Hospital of Soochow University, Changzhou, Jiangsu, China

**Keywords:** hepatocellular carcinoma, two-dimensional materials, tumor diagnosis, dynamic therapy, review

## Abstract

In recent years, two-dimensional (2D) layer materials have shown great potential in the field of cancer diagnosis and treatment due to their unique structural, electronic, and chemical properties. These non-spherical materials have attracted increasing attention around the world because of its widely used biological characteristics. The application of 2D layer materials like lamellar graphene, transition metal dichalcogenides (TMDs), and black phosphorus (BPs) and so on have been developed for CT/MRI imaging, serum biosensing, drug targeting delivery, photothermal therapy, and photodynamic therapy. These unique applications for tumor are due to the multi-variable synthesis of 2D materials and the structural characteristics of good ductility different from microsphere. Based on the above considerations, the application of 2D materials in cancer is mainly carried out in the following three aspects: 1) In terms of accurate and rapid screening of tumor patients, we will focus on the enrichment of serum markers and sensitive signal transformation of 2D materials; 2) The progress of 2D nanomaterials in tumor MRI and CT imaging was described by comparing the performance of traditional contrast agents; 3) In the most important aspect, we will focus on the progress of 2D materials in the field of precision drug delivery and collaborative therapy, such as photothermal ablation, sonodynamic therapy, chemokinetic therapy, etc. In summary, this review provides a comprehensive overview of the advances in the application of 2D layer materials for tumor diagnosis and treatment, and emphasizes the performance difference between 2D materials and other types of nanoparticles (mainly spherical). With further research and development, these multifunctional layer materials hold great promise in the prospects, and challenges of 2D materials development are discussed.

## 1 Introduction

Two-dimensional (2D) layer materials are a class of nanomaterials with unique structural properties with a relatively novel way of arranging the atoms of a material in a single layer (Campi et al., 2023; Chitara et al., 2023; Sierda et al., 2023). These materials with special morphology are often composed of layer structures and transition metal sulfides or single elements with thicknesses that range from several atoms to a few nanometers (Chen et al., 2022; He et al., 2023; Hou et al., 2023). Based on their element composition and spatial distribution, their range of applications includes but is not limited to nanoelectronics, photonics, and biomedicine (Li X. et al., 2023; Liu et al., 2023a; Yu et al., 2023a; Huang et al., 2023). In the context of biomedicine, 2D layer materials have the potential to revolutionize the way that clinical doctors diagnose, monitor, and treat diseases. Due to the significant physicochemical advantages and solution stability of these materials, they can easily be widely used in various biosensor components and drug delivery fields (Geng et al., 2023; Luo et al., 2023).

Compared with ordinary spherical particles, 2D layer materials have quite different physical, chemical and biological properties (Singh A. et al., 2023; Wang et al., 2023a; Yu et al., 2023b; Zhang et al., 2023). First of all, intuitively speaking, 2D materials have a great difference in thickness scale and long and wide scale, which is determined by the growth or stripping mode of the lamellar structure. Secondly, considering the larger specific surface area and more electron holes, 2D materials are often more suitable for surface loading of drugs and photothermal and photoelectric conversion. In addition, in terms of fluid mechanics, 2D materials show special motion rules that are different from ordinary spherical materials, which makes navigation and targeting in human tumor blood vessels possible.

**FIGURE 1:**
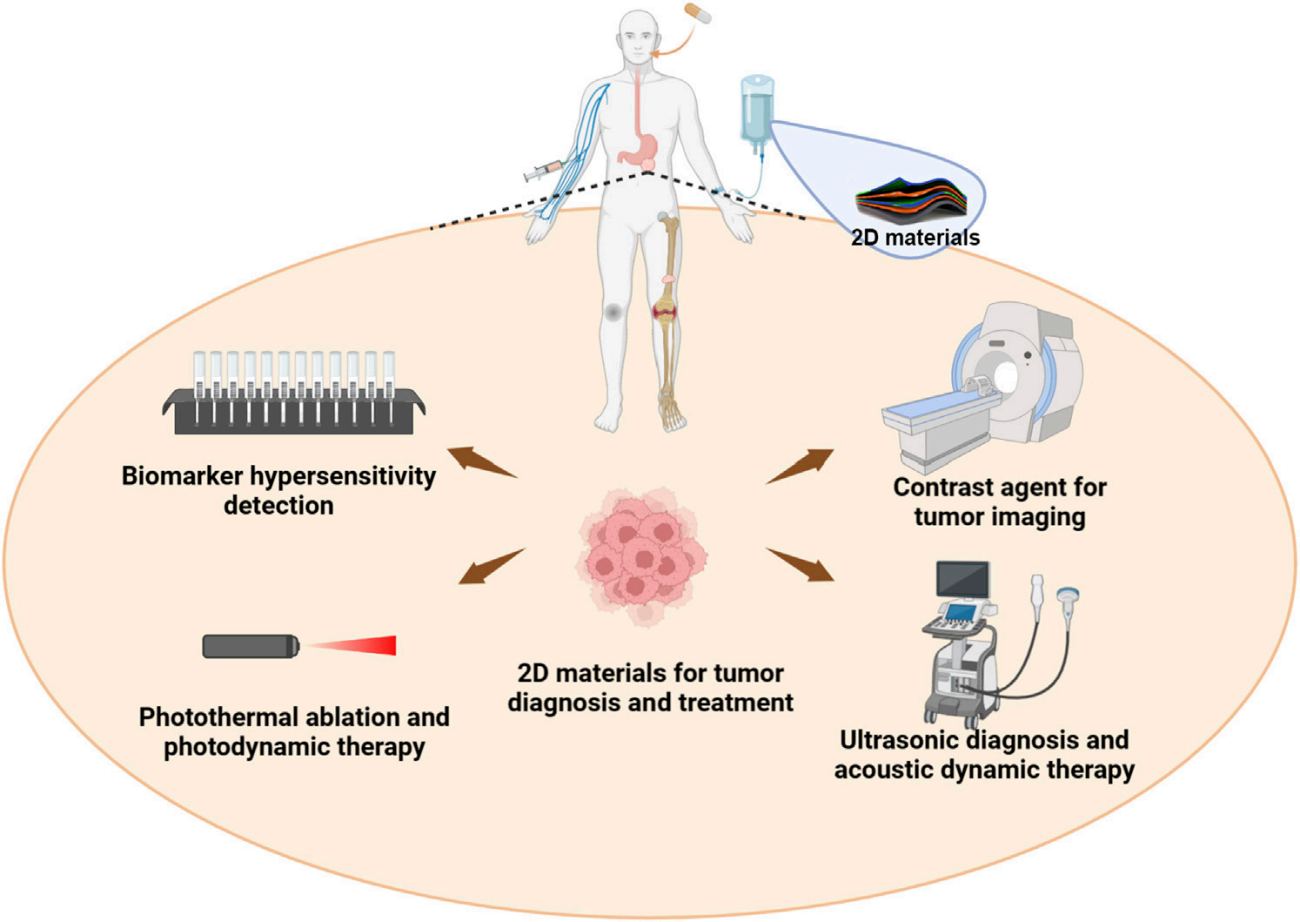
The multidisciplinary biomedical and cancer therapeutic applications of two-dimensional layer materials including efficient diagnosis of serum markers, MRI/CT imaging diagnosis, near-infrared light based ablation and dynamic therapy, ultrasound-based diagnosis and dynamic therapy, and the combination of various therapies. The diagram shows not only the multiple drug administration mode at the top, but also the various diagnosis and treatment methods involved in 2D materials at the bottom.

Although serum screening and imaging analysis have aided in the diagnosis of cancer, the personalized diagnosis of cancer remains one of the most challenging diseases to diagnose and treat (Li R. et al., 2022; Kinker et al., 2023; Minton, 2023). The main development bottleneck is that the sensitivity of serum tumor markers needs to be improved, and the clarity and discriminability of images need to be improved. Because early detection of cancer is critical for successful treatment, there has been much progress in the development of new imaging techniques to achieve this, such as magnetic resonance imaging contrast medium (MRI), low-dose computed tomography (CT), and fluorometric positron emission tomography (PET) (Karlsson et al., 2023; Lawrence et al., 2023; Lee et al., 2023). In addition, targeting therapies such as immunotherapy and intracellular kinase inhibitors have revolutionized cancer treatment (Hilton et al., 2023; Lin et al., 2023; Roy et al., 2023). These strategies are designed to specifically target cancer cells, and minimize damage to healthy cells to enhance clinical outcomes. Based on the above-mentioned directions of tumor diagnosis and treatment, 2D materials have had relatively important development and progress in the above fields in the past period of time (Liu et al., 2023b; Brevi and Zarrinpar, 2023; Kong et al., 2023; Meng et al., 2023) (Figure 1). In this review, we will provide an updated overview of the latest advancements in the application of 2D layer materials in tumor diagnosis and treatment, from a perspective that combines both fundamental research and hopeful clinical translation (Hu et al., 2023).

## 2 Varieties, structure and characterization of 2D materials

In terms of elemental composition, ultra-thin and even atom-thick 2D material sheets with almost only a single atom thickness can be divided into: graphene, transition metal sulfides, metal nitrides, double hydroxide, etc. Under the condition of their unique microscopic morphology, their important microscopic characteristics are their specific surface area and photoelectric properties. Two-dimensional materials that can be applied in the field of cancer systematic therapy generally meet the requirements of small individual lamellar layers, large specific surface area, pore size or potential hydrogen bonds for drug loading, and electron holes for photothermal and dynamic therapy.

Due to the special properties of the crystal structure, 2D materials show different electrical or optical properties of anisotropy, including Raman spectrum, photoluminescence spectrum, second harmonic spectrum, light absorption spectrum, thermal conductivity, electrical conductivity and other properties of anisotropy. These photoelectric signals and energy conversion, which are different from human tissues and blood, are needed for cancer diagnosis and treatment. At the same time, these unique physical and chemical properties can help the layers in the blood to get rid of the disorderly Brownian motion and gather to the tumor site through the EPR effect.

The main types of 2D materials used in the field of biosensors and cancer diagnosis and therapy are listed in Table 1. Compared with traditional spherical nanoparticles, such materials tend to have a simpler and more uniform distribution of elements, as well as a more stable electron transport efficiency in solution. This makes their application scenarios often focused on, but not limited to, photothermal or photodynamic energy conversion as well as biomolecular enrichment and signal amplification. This may be the reason why 2D materials are widely used and constantly updated in the field of cancer diagnosis and treatment. In addition, in the field of material modification, 2D material surface modification has been widely paid attention to, its main purpose is to increase its drug load rate and biocompatibility in aqueous solution.

**TABLE 1:** Summary of the types, basic characterization and application directions of 2D materials in the field of cancer diagnosis and treatment.

No.	Material type	Element composition	Size scale	Biological effect	Ref
1	P6m lattice	Dihedral protein	31 nm	Endocytic block	Ben-Sasson et al. (2021)
2	MBene	zirconium boride	∼50 nm	NIR chemothermal and gasothermal therapy	Chen et al. (2021)
3	Graphene oxide	GO-AgInS2	∼500 nm	Interaction with monocytes and B cells	Orecchioni et al. (2020)
4	Selenium-coated tellurium nanoheterojunction	TeSe	∼33 nm	PTT, inducing tumor cell death and alters TME	Chen et al. (2020)
5	MXene	Ti3C2/AuNPs/SPA	40 nm–1 μm	High specificity for CEA in real serum samples	Wu et al. (2019)
6	MXene	Co3O4, Ti3C2Tx	50 nm–1 μm	Rapid detection of H2O2 for clinical diagnostics	Singh et al. (2023b)
7	2D Monoelemental Germanene Quantum Dots	GeQDs	4.5 nm	Power-dependent photothermal effects	Ouyang et al. (2019)
8	2D Bi/BiOx Lateral NanoHeterostructure	Bi/BiOx	55 nm	PDT suppressed by the hypoxic tumor microenvironment with ROS generation	Qiu et al. (2021)
9	Carbon/potassium-doped red polymeric carbon nitride	C3H6N6, KCl	50–200 nm	NIR photocatalysts for hydrogen therapy	Xu et al. (2021)
10	2D Multienzyme-Mimicking Pyroptosis Inducers	NiCoOx	178 nm	Ultrasound-augmented catalytic tumor nanotherapy	Song et al. (2023a)

After careful comparison, 2D materials are designed as ultra-thin substances composed of just one or a few atomic layers in a flat structure. They possess exceptional properties like high flexibility, large surface-to-volume ratio, and excellent electrical conductivity. In contrast, microspheres are small spherical particles with diameters ranging from micrometers to nanometers. As a result, microspheres are better encapsulated and therefore more suitable for slow release of drugs, but the problem is that they require a specific release environment and relatively low load rate (Li C. et al., 2023; Song Z. et al., 2023). Microspheres have unique features like controlled size, porosity, and surface properties. In summary, 2D materials are atomically thin with remarkable electronic properties, while microspheres are three-dimensional particles with controlled characteristics, and they constitute important macromolecular drug loads and tumor therapeutic substances at micro scale.

## 3 Sensitive serum screening and imaging cancer diagnosis

Screening and diagnosis of cancer depend on rapid, sensitive screening of serum and imaging responses including but not limited to ultrasound, MRI and CT images (Ahn et al., 2018; Gao et al., 2021; Yan et al., 2021). In addition, 2D layer materials absorb and emit light in the visible and near-infrared (NIR) range, which overlaps with the spectral window for biological tissues (Kakkar et al., 2022; Kim et al., 2022; Selvaggio and Kruss, 2022). This allows for deeper penetration of light into tissues, enabling high-resolution imaging of tumors deep inside the body. Furthermore, 2D layer materials possess relatively high photostability, which enables long-term imaging without the need for continuous re-administration of contrast agents (Koo et al., 2021; Huang et al., 2022; Jayachitra et al., 2022). The main advantages of 2D materials in cancer imaging are their high degree of biocompatibility and bioactivity, as well as excellent optical and magnetic properties.

**FIGURE 2:**
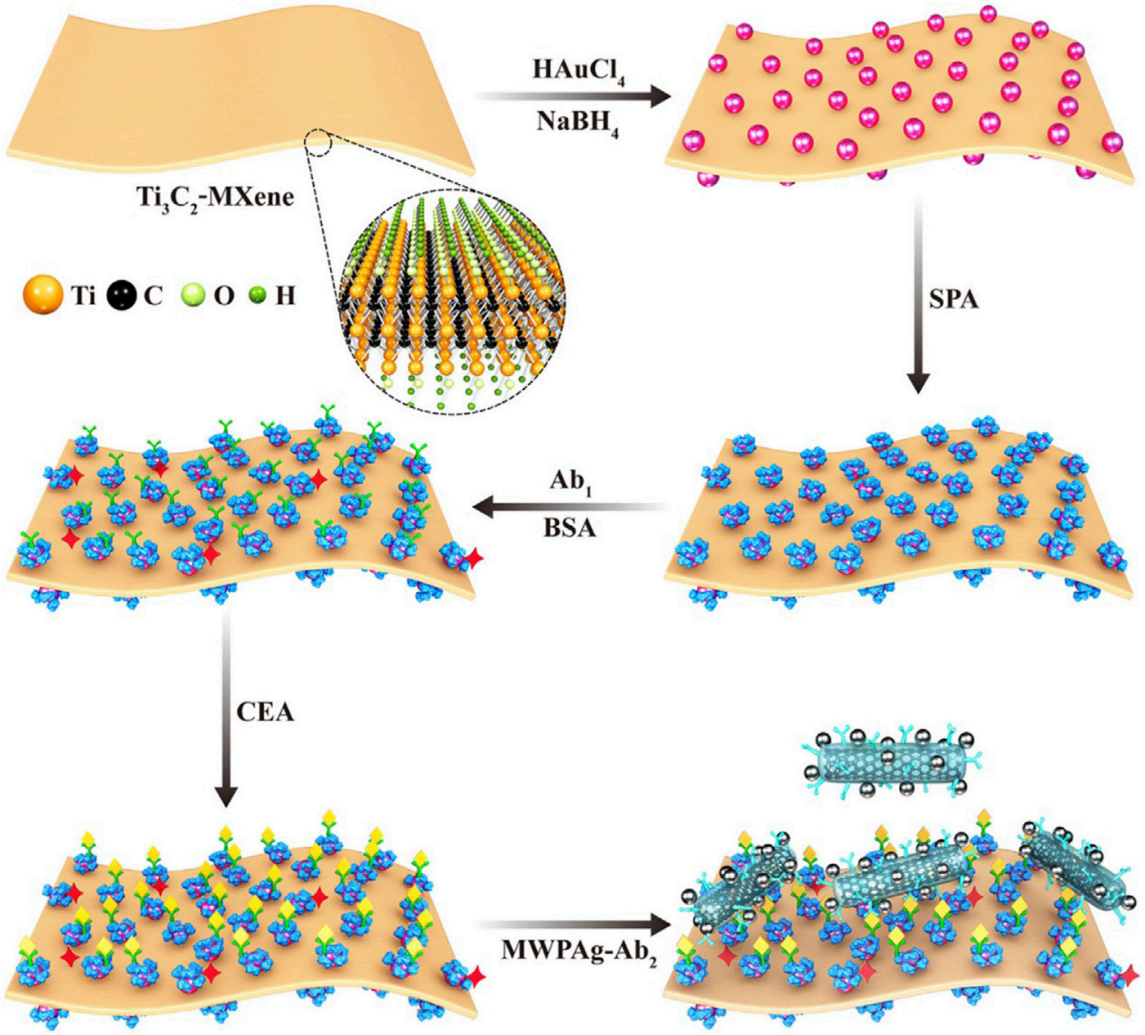
Detection of serum CEA using MXene as biosensor. Reproduced from Wu et al. (2019) with permission Copyright 2022, American Chemical Society.

Serum tumor markers play an important role in screening and predicting tumor recurrence and treatment prognosis in healthy people. More representative is the surface plasmonic resonance (SPR) technique, a new serum test item that has become an *in situ* bioaffinity detection technology without fluorescence or enzyme labeling. Wu et al. (2019) proposed using Ti3C2-MXene/AuNPs/SPA as a biosensor platform to improve the detection sensitivity of serum markers (Figure 2). The Ti3C2-MXene biosensor is highly sensitive to CEA biomarkers and has good selectivity, reproducibility and recovery in human serum samples. The differences of 2D materials from microsphere in the detection of cancer serum markers are their high sensitivity, high selectivity and fast response speed, which help to improve the accuracy and efficiency of early cancer diagnosis.

**FIGURE 3:**
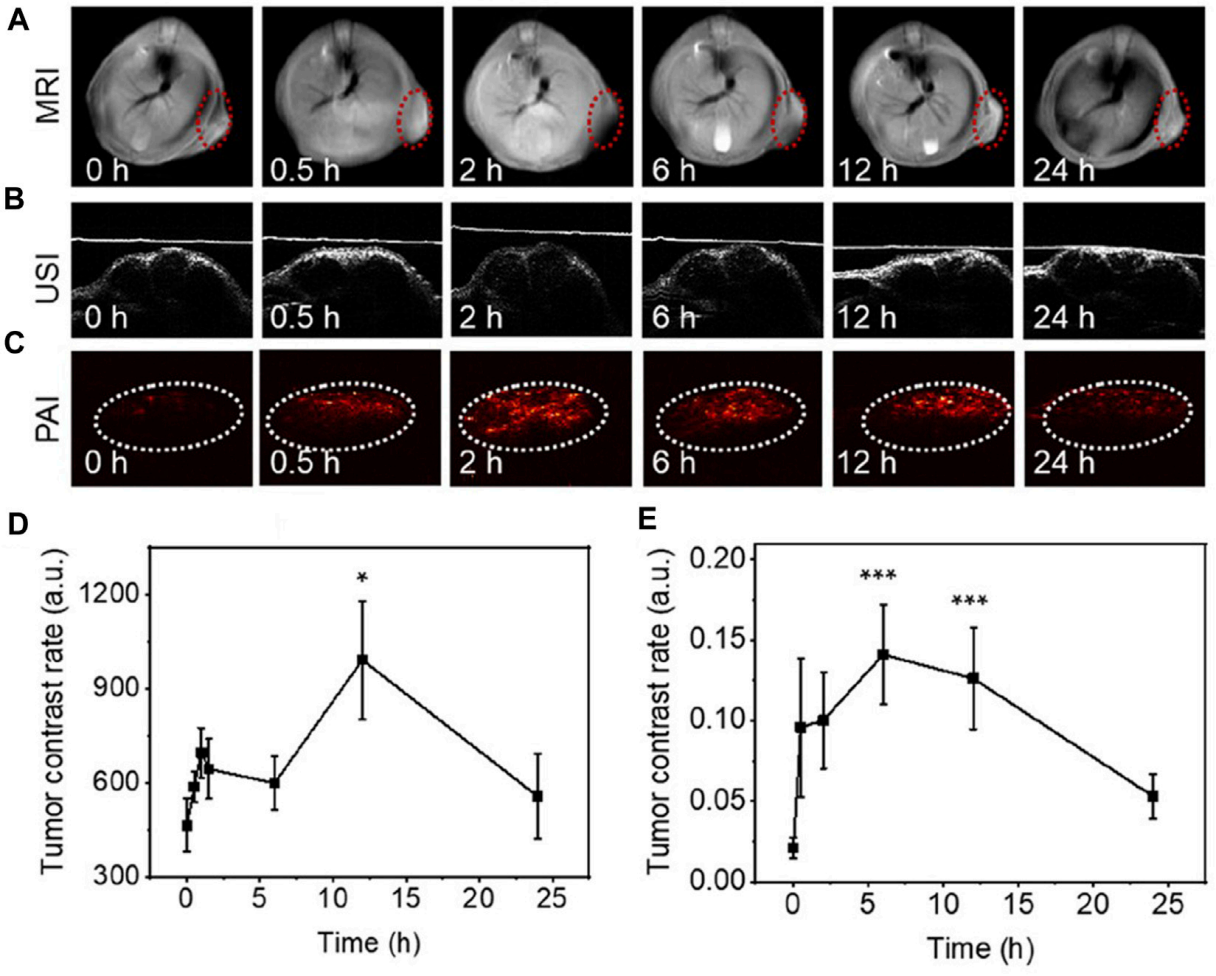
MRI and PAI imaging *in vivo* based on 2D carbon nanochips containing ferric ions. **(A–C)** Distribution of nanochips at tumor sites over time. **(D, E)** Correlation quantitative recording of MRI and PAI signals. Reproduced from Yin et al. (2022) with permission Copyright 2022, American Chemical Society.

As a non-invasive and radiation-free tissue image analysis method, MRI is playing an important role in the diagnosis and staging evaluation of various cancers. The 2D material has been found to be useful as an enhanced developer for MRI or similar harmless imaging tests without causing damage to normal tissue. Yin et al. (2022) created a nanohybrid based on carbon nanochips that not only achieved the synergistic effect of photothermal therapy and chemodynamic therapy to inhibit the extremely rapid growth of RM1 tumors in mice, but also allowed photoacoustic and magnetic imaging, guided drug delivery and IR-II irradiation imaging therapy (Figure 3). These materials also exhibit highly tunable and specific surface coatings, which allow for the conjugation of various biomolecules such as peptides, antibodies, and tumor-targeting ligands. Such strategies provides the possibility of achieving highly targeted and sensitive imaging of tumors.

Benefiting from the large comparative area and surface electron holes of the 2D material, it can load more contrast agent to the cancer area without being prematurely degraded in the blood. This large surface area allows for high drug-loading capacity, meaning that drugs can be delivered directly to tumors for therapy while simultaneously being used as a contrast agent for imaging. There are several examples of 2D layer materials that have been studied for their potential as tumor imaging agents. Among them, graphene oxide (GO) and transition metal dichalcogenides (TMDs) such as molybdenum disulfide (MoS2), and tungsten disulfide (WS2) are the most widely studied (Zhu et al., 2020; Miao et al., 2021; Truong Hoang et al., 2023; Xia et al., 2023). Graphene oxide is an oxygenated form of graphene that exhibits excellent biocompatibility and facile surface functionalization. GO has been used as a contrast agent for various imaging modalities, including fluorescence imaging, photoacoustic imaging, and MRI. For example, GO nanocomposites incorporating gadolinium (Gd) or iron oxide (Fe3O4) have been demonstrated with high contrast for MRI imaging (Jiang et al., 2021). The outstanding contribution to magnetic resonance imaging makes 2D materials outstanding in harmless imaging tests, and can be repeatedly and safely used for cancer detection and screening diagnosis.

**FIGURE 4:**
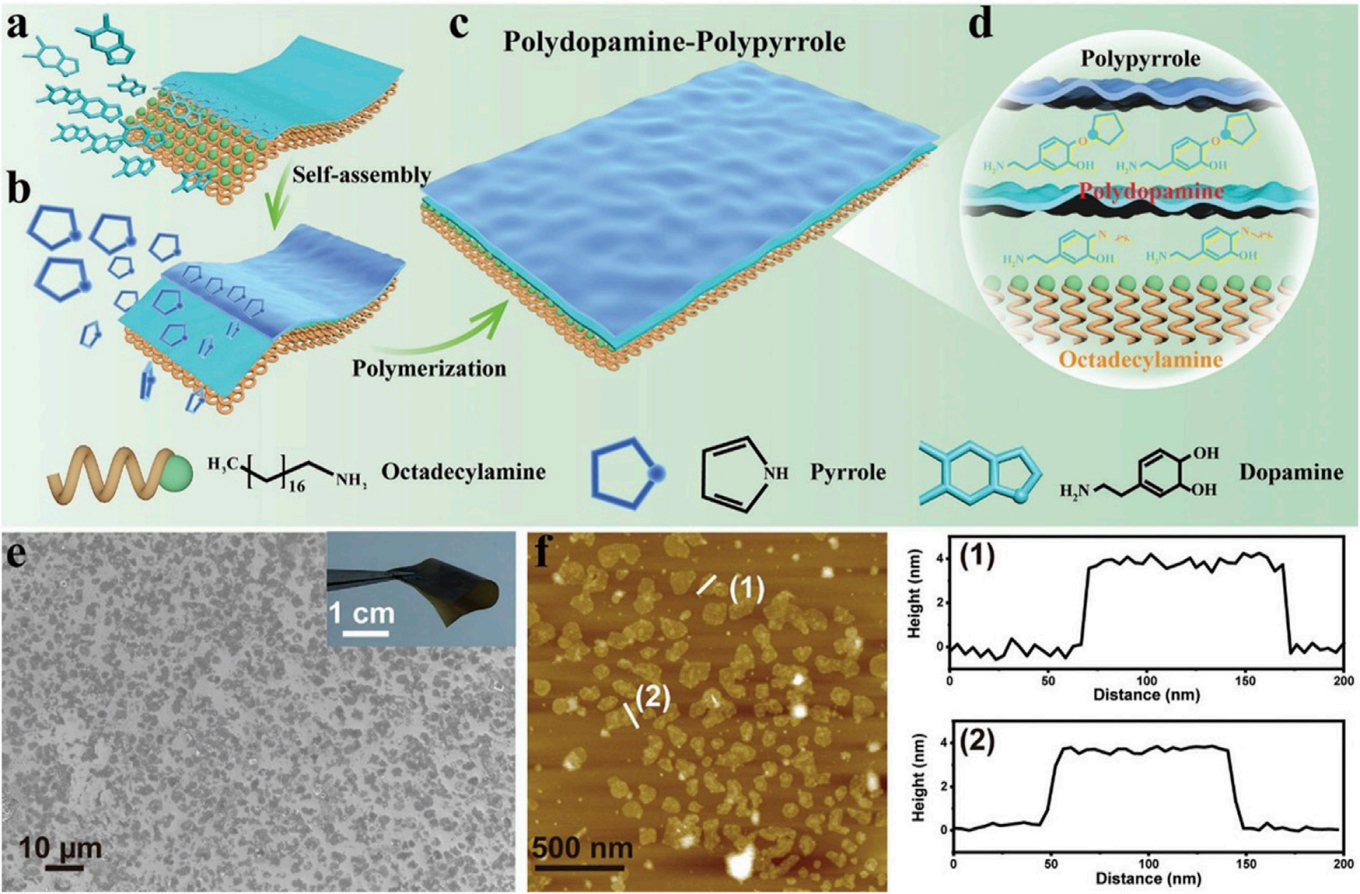
2D DPPy nanosheets for PA imaging contrast agents. **(A–D)** A schematic illustration shows the synthesis of a nanoparticle based on polydopamine. **(E, F)** SEM images of DPPy nanosheets and the corresponding height profiles. Reproduced from Geng et al. (2023) with permission Copyright 2023, Wiley.

In addition to the above imaging methods based on magnetic resonance, 2D materials have also made many advances in the analysis methods based on CT and different wavelengths of light and rays. For example, TMDs have excellent photoluminescence properties, making them a suitable fluorescent imaging agent. Furthermore, they have been utilized for magnetic resonance and X-ray computed tomography imaging (D'Agosta et al., 2023; Singh et al., 2023c). In addition to GO and TMDs, other 2D layer materials such as black phosphorus (BP), boron nitride (BN) and metal-organic frameworks (MOFs) have also been studied for tumor imaging (Li M. et al., 2023; Ra et al., 2023; Zheng et al., 2023). BP has a high photoluminescence quantum yield and excellent NIR imaging properties. BN and MOFs, on the other hand, have the unique ability to bind and release therapeutic agents onto cancer cells and have been utilized to deliver photothermal therapy in cancer treatment. Geng et al. (2023) applied two-dimensional therapeutic nanosheets composed of PD and PPy through a simple self-assembly method (Figure 4). The introduction of PD and PPy makes two-dimensional nanostructures with high biocompatibility, flexibility and stability. Although it is difficult to maintain long-term stability in organisms, 2D materials can form short-term stable states that are more conducive to drug release and transport through covalent bonds or hydrophilic and hydrophobic interactions with small molecules and polymers.

CT is a widely used imaging modality that utilizes X-rays to produce a series of cross-sectional images of the body. While it has high spatial resolution, CT has limited sensitivity in detecting tumors, particularly small and early-stage tumors (Liu et al., 2023c; Tisi et al., 2023). 2D layer materials such as GO and TMDs have been studied as contrast agents to improve the sensitivity of CT imaging. In a study by Li X. et al. (2022) safe and efficient platform of TOS married MoS2 was utilized as a nanoparticulate CT contrast agent that improved the sensitivity and contrast in CT imaging of early-stage tumors in mice. Similarly, in a study by Popescu et al. (2023) IONP-DOX functionalized with a tumor-targeting peptide were used as a CT contrast agent for highly specific tumor imaging in a mouse model. Overall, 2D layer materials have the potential to revolutionize tumor imaging, with their unique physicochemical and optical properties enabling precise and sensitive tumor detection across various imaging modalities.

**FIGURE 5:**
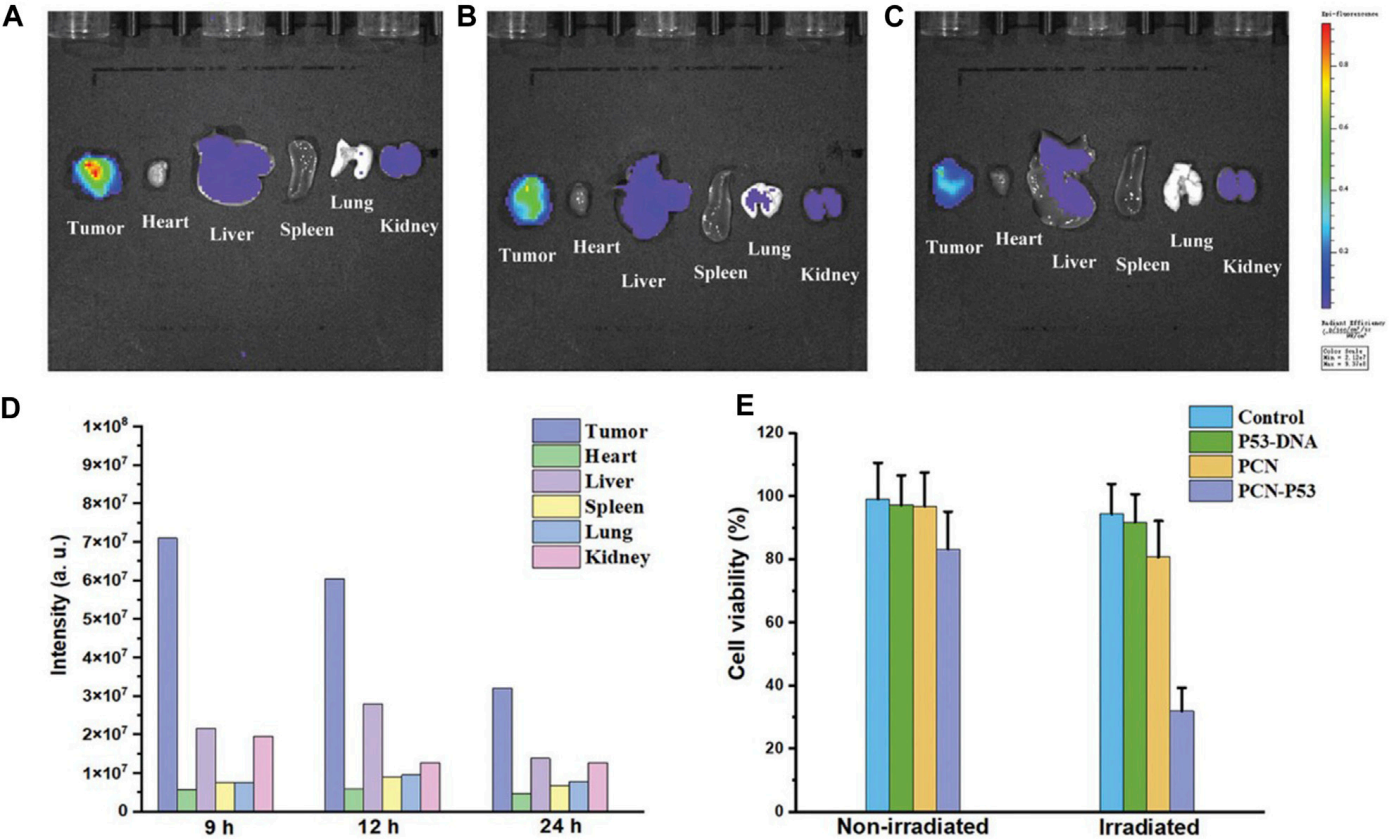
*In vivo* imaging of a nude mouse tumor model after injection of PCN-DNA. **(A–C)** Comparison of tumor fluorescence signals and internal organs at different time points (9, 12, and 24 h). **(D,E)** Corresponding signal strength quantitative results. Reproduced from Liu et al. (2022) with permission Copyright 2020, Wiley.

Fluorescence imaging is widely used for tumor imaging due to its high spatial resolution and sensitivity (Chen et al., 2023; Izquierdo-Garcia et al., 2023; Wu et al., 2023). 2D layer materials such as graphene and TMDs are fluorescent and can be functionalized to target tumor-specific receptors (Behera et al., 2022). This has led to the development of highly sensitive and specific imaging probes for tumor imaging. For example, in a study by Liu et al. (2022), PCN nanosheet was functionalized with enough ROS under light conditions, and the process of gene delivery can be tracked in real time through fluorescence imaging technology. Similarly, TMDs such as MoS2 have been used as fluorescent imaging agents. In a study by Ying et al. (2020) raised a method for the specific determination of AMACR in real human serum using electrochemical microsensor system with MoS(2) film surface (Figure 5). In order to implement the protocol, several self-organized nanohybrid material consisting of metal nanocolumns was developed in a two-dimensional MoS(2) matrix as the sensing interface material (Wang X. et al., 2020; Ying et al., 2020). The targeting MoS2 quantum dots demonstrated high specificity and sensitivity in imaging tumor in a mouse model. Its high fluorescence quantum yield, tunable emission wavelength and excellent biocompatibility help to improve the sensitivity and accuracy of cancer diagnosis.

In summary, 2D layer materials offer several advantages, including element diversity, high sensitivity, specificity, low toxicity, and precise targeting, making them ideal candidates as contrast agents for tumor imaging. With continued advancements and development of these materials, they hold significant promise for revolutionizing tumor diagnosis and treatment. Firstly, due to the diversity of elements, 2D materials can be widely used in CT and MRI imaging experiments, and their imaging principles are also diversified. Secondly, 2D layer materials possess physicochemical properties such as high photostability, excellent biocompatibility, and NIR optical properties, making them suitable for imaging deep inside the body without causing toxicity. Additionally, 2D layer materials have a large absorption cross-section, resulting in strong light-matter interactions leading to high contrast imaging. Another advantage of 2D layer materials is their highly tunable nature. They can be easily synthesized, scaled up, and surface-functionalized to provide high-targeting specificity for tumor imaging. In contrast, traditional imaging agents are limited in their multifunctionality and require complex synthesis processes and may not provide specificity in targeting tumors.

## 4 Cancer inhibition with energy conversion and dynamic therapy

2D materials are widely used in energy conversion therapy and kinetic therapy of cancer because their carrier migration and heat diffusion are confined to two-dimensional plane. Among them, the most representative is photothermal ablation therapy, which plays the role of thermal ablation by increasing the heat of the local area of the tumor by nearly infrared red light (Wang et al., 2023b; Li Y. et al., 2023; Zhou et al., 2023). In addition to inducing the direct necrosis of tumor cells, it can also guide the programmed apoptosis of cells and the change of tumor microenvironment. 2D layer materials such as graphene, gold nanorods, and black phosphorus have been studied for their potential as photothermal therapy agents.

### 4.1 The energy conversion represented by the photothermal effect

**FIGURE 6:**
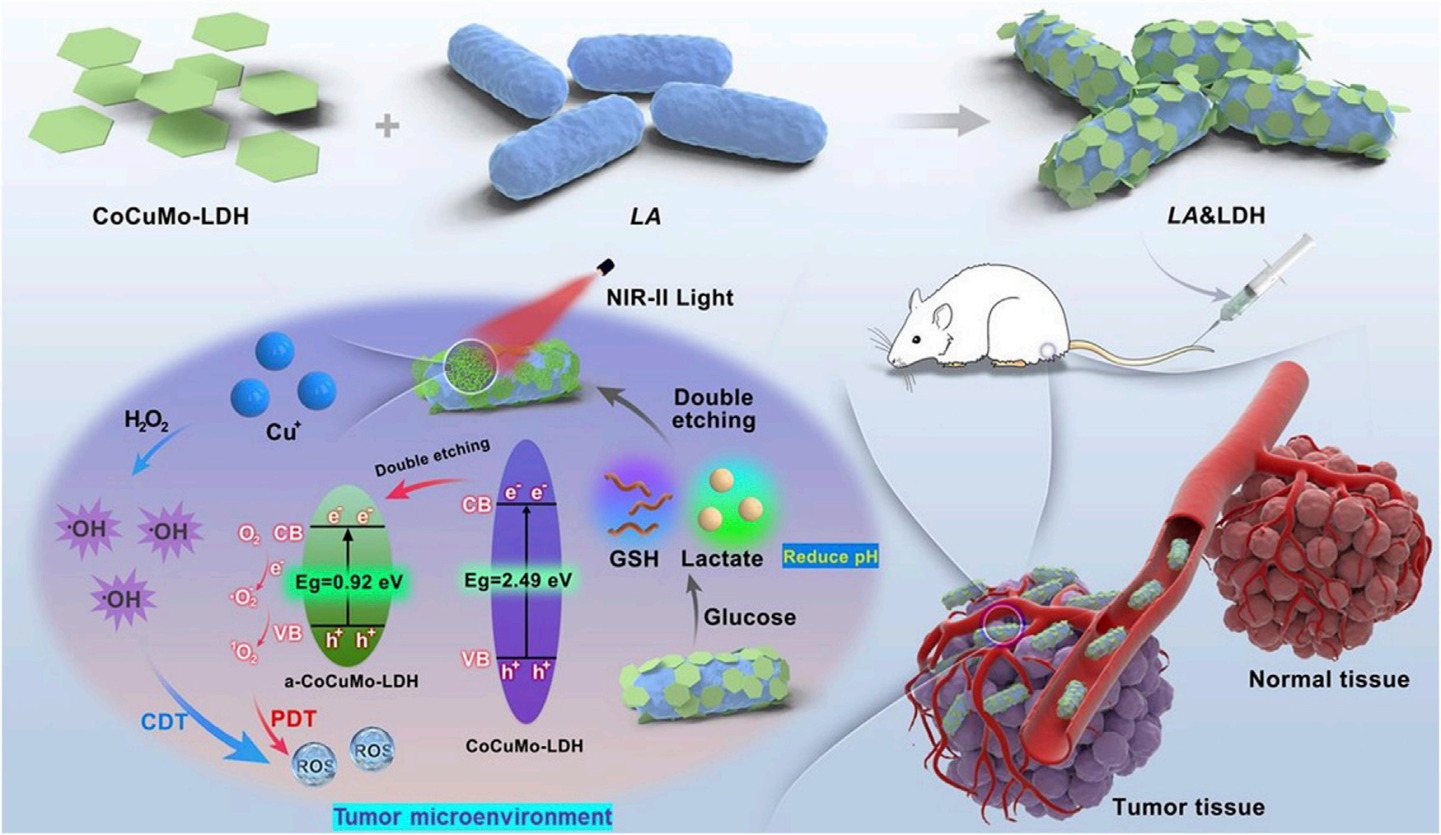
Schematic illustration of NIR-II photodynamic combination therapy using LA&LDH *in situ* activated by TME for tumor targeting. Reproduced from Yang et al. (2023) with permission Copyright 2023, Wiley.

Recent advancements in the synthesis and functionalization of 2D layer materials have enabled the development of more precise, efficient, and selective targeted therapy techniques to improve light-heat conversion efficiency (Liu B. et al., 2023; Liu et al., 2023e; Sutter and Sutter, 2023). By appropriate lamellar size stripping and surface molecular modification, 2D materials were effectively converged to the tumor site, albeit intravenously, followed by corresponding ablative therapy under direct vision. Such targeting effect mainly depends on receptor ligand binding and EPR effect. Recently, many studies have used the homologous compatibility principle of biofilm to enhance the micro-scale active targeting effect. For instance, 2D layer materials such as CoCuMo nanosheet have been conjugated with *lactobacillus* acidophilus (LA), enabling selective targeting of cancer cells over healthy cells (Yang et al., 2023) (Figure 6). LA acts as a biological carrier of CoCuMo and a combined photothermal and photodynamic medium. Due to the large differences in the horizontal scale of 2D materials, it can be used as a microbial load or bionic application, through the modification and alteration of its surface. This allows carriers such as bacteria and cells to deliver internal biomacromolecules or plasmids directly to the tumor site without destroying their cell membranes.

### 4.2 Kinetic therapy and biological reactive oxygen species

**FIGURE 7:**
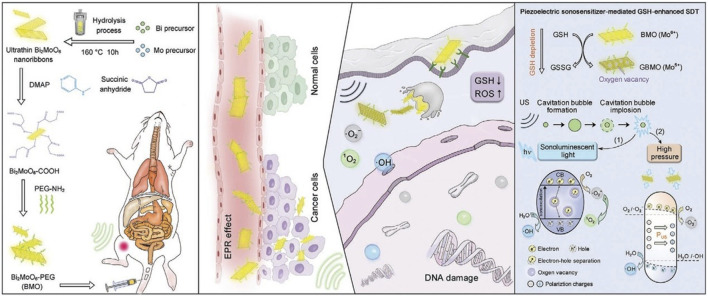
Schematic diagram of two-dimensional piezoelectric Bi2MoO6 acoustic sensitizer for GSH enhanced sonodynamic therapy, including synthesis process, mechanism of action *in vivo* and kinetic reaction. Reproduced from Dong et al. (2021) with permission Copyright 2021, Wiley.

In addition, 2D layer materials have be activated by external stimuli, such as light, magnetic fields, ultrasound, or pH changes, to release their payload of drugs stimulating sonodynamics and chemical dynamics in addition to photodynamics mentioned. *In vivo*, conversion reactions with singlet oxygen and peroxanions become particularly active because 2D materials provide a wide range of reaction areas and electron holes. Dong et al. (2021) successfully performed endogenous GSH enhanced sonodynamic therapy with a piezoelectroacoustic sensitizer (Figure 7). The design of the piezoelectric BMO sensor demonstrates the enhanced e-−h+ separation by endogenous gsh and the accelerated ROS generation by exogenous US, where the energy of US can be maximized to obtain excellent treatment results. This actually combines chemokinetic therapy and sound dynamic therapy.

**FIGURE 8:**
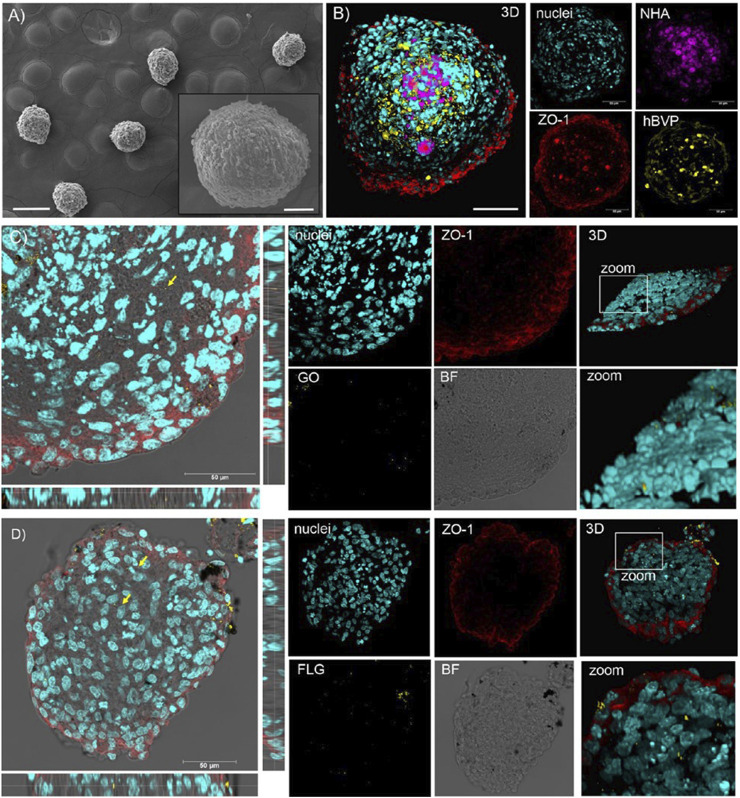
Scanning electron microscopy and confocal microscopy analysis of the interaction of graphene oxide and FLG with a three-dimensional multicellular assembly model of the human blood-brain barrier. **(A, B)** SEM and confocal images of hMCA show the morphology and 3D reconstruction. **(C, D)** 20μm hMCA slices incubated with 10 μg/ml graphene oxide or FLG were representative of confocal XYplanes, zprojection, and 3D reconstruction. Reproduced from Castagnola et al. (2023) with permission Copyright 2023, American Chemical Society.

In order to transport itself through tissue spaces and vascular basement membranes to deliver drugs to tumor sites, 2D materials have to be engineered differently for different biological barriers. In a study by Castagnola et al. (2023) the combination of GO with FLG demonstrated increased blood-brain barrier (BBB) permeability (Figure 8). This approach ensures that the drug is released only by specific stimuli provided by the 2D layer materials, reducing the risk of off-target side effects. Another advancement in 2D layer materials for targeted therapy is their ability to penetrate the blood-brain barrier (BBB) (Wolf et al., 2019; Maleki et al., 2021; Wang L. et al., 2023). The BBB is a highly selective barrier that limits the entry of molecules into the brain, hindering the effectiveness of treatments for brain tumors. In terms of particle size, 2D materials are not suitable for passing directly through the BBB, but they can increase the concentration of drugs passing through the BBB through location-based release and anchoring degradation.

### 4.3 Immune adjuvants and immune activation

**FIGURE 9:**
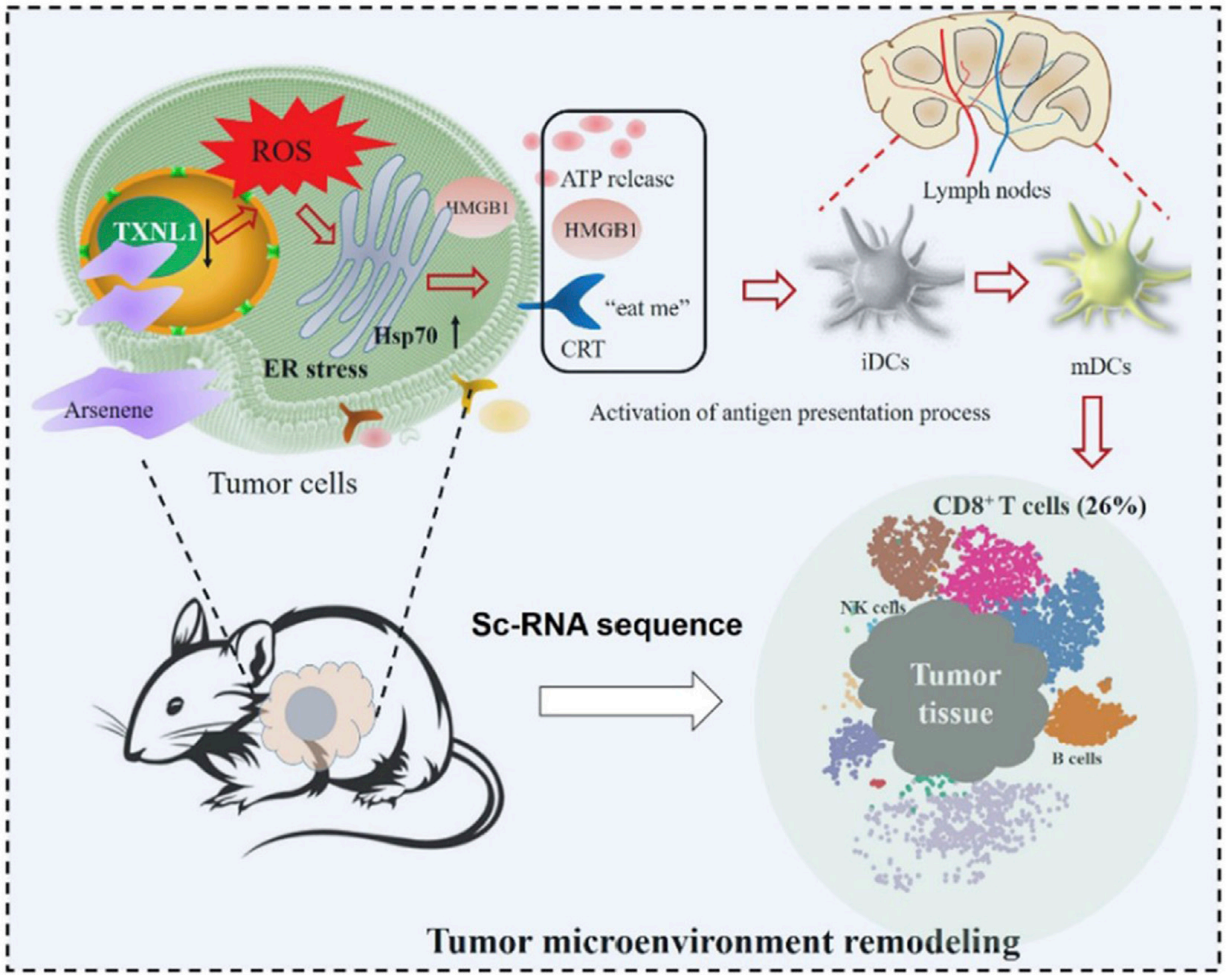
The immunomodulatory processes of arsenic in cancer cells and tumor microenvironments were mapped by proteomic analysis and scRNA sequencing analysis, respectively, revealing the unexpected immunomodulatory potential of arsenic nanosheets. Reproduced from Wang et al. (2022) with permission Copyright 2022, American Chemical Society.

In order to overcome drug tolerance and tumor recurrence caused by physicochemical therapy and small molecule drug delivery, stimulate long-term immunity and improve tumor microenvironment, immunotherapy-related biomacromolecules were introduced into the design of 2D materials. By targeting originally inhibitory immune cell receptors, immunotherapy regulates the local and systemic immune status of tumors and the degree of invasion of antigen-presenting cells and T cells (Liu et al., 2021; San Emeterio et al., 2021; Nie et al., 2022). Wang et al. (2022) analyzed *in vivo* single cell RNA sequencing and tumor cell proteomics and the mechanism of action of arsenene, and the unexpected immunomodulatory ability of arsenic was found (Figure 9). By analyzing the related effects of nano-sheet itself on tumor immunity, it can guide the target selection of immunotherapy and further clinical transformation. In addition, 2D layer materials can be utilized as a delivery platform for immune-stimulating agents such as cytokines, interferons, and interleukins. These immunoregulatory agents can be encapsulated within the layers or conjugated to the surface of 2D layer materials to increase their efficacy.

In addition, layered double hydroxide (LDH) as a representative of inorganic 2D inorganic materials, has a unique potential for tumor diagnosis and treatment. It has stronger anion conversion ability and better thermal stability than traditional 2D materials, and is considered as a potential carrier in the field of tumor energy therapy. One typical study used LDH as a carrier for CoFe photothermic agents, resulting in better energy conversion and delivery within tumors (Wang L. et al., 2020). In addition, another study showed that LDH can support DOX systematic therapy, showing good adhesion and antimicrobial properties, and this PH-dependent release mechanism is conducive to accurate treatment of tumor sites and the prevention of related infection complications (Kiani et al., 2022).

Overall, 2D layer materials offer a unique set of properties that can be leveraged for targeting tumor therapy. By ensuring high drug-loading capacity and selective delivery of drugs to cancer cells, 2D layer materials hold significant promise in advancing targeted therapy approaches for cancer treatment (Xia et al., 2021). The energy conversion and electron transport involved mainly involve but are not limited to photothermal ablation, photodynamic, sonodynamic, chemical dynamic and immunomodulatory therapy. Additionally, 2D layer materials could be a multimodal platform that allows for the combination of multiple therapies for optimal treatment outcomes. With further research, it is expected that 2D layer materials will play a significant role in revolutionizing cancer treatment.

## 5 Future perspectives and challenges

Personalized medicine is an approach that tailors medical treatment to the individual patient’s needs based on their individual genetic makeup, lifestyle, and environment (Ben-Akiva et al., 2023; U.S.P.S.T. Force et al., 2023). 2D layer materials offer an ideal platform for personalized medicine due to their precise targeting capabilities and customization of surface functionalization (Bhakat et al., 2023; Li W. et al., 2023; Perez et al., 2023). This class of materials plays an important role in the fields of serum marker screening, imaging diagnosis and energy conversion and ROS-based therapy. Compared to traditional spherical nanoparticles, 2D materials have an excellent advantage in terms of surface modification and loading, as well as rapid electron transferring. Furthermore, 2D layer materials hold great potential in real-time tracking of therapeutic response. In the modern medical context where cancer treatment plans need to be adjusted at any time according to the progress of the disease, this diversified diagnosis and treatment model can effectively help determine the individual plan.

At present, the main obstacles in clinical conversion practice of 2D materials are simple synthesis processes and the need for stability of material size and dispersion in aqueous solutions (Azam and Mahjouri-Samani, 2023; Koner et al., 2023; Shen et al., 2023). The synthesis of 2D layer materials is a complex process, and the ability to scale up production while maintaining quality and consistency is critical. Because most 2D materials themselves contain exogenous substances recognized by the immune system and complement system, they may cause abnormal thrombosis and vascular damage, despite more modifications on surface. This is also one of the main problems to be overcome in the future application direction of inorganic materials and organic small molecule biology. Moreover, the development of regulatory guidelines for 2D layer materials for medical applications is also necessary. It is essential to establish safety and efficacy guidelines for the use of 2D layer materials in cancer treatment to ensure their translation from bench to clinics through rigorous clinical trials and human evaluations.

In summary, the strategies of effective tumor therapy and imaging mediated by 2D materials may provide a shortcut for the clinical application of nanomaterials. Despite many difficulties and challenges, clinical research is always in need of new and highly effective treatment strategies to complement and improve efficacy. In the future biological application of anisotropic high specific surface area materials represented by 2D materials, there will be more particles suitable for drug delivery as well as tumor diagnosis and comprehensive treatment. 2D layer materials will also gradually make greater progress and development from design synthesis to modification characterization to biological application and transformation.

## 6 Conclusion

In conclusion, 2D layer materials have emerged as a promising new frontier in cancer diagnosis and treatment. With their unique properties, including a high surface area, multifunctionality, and customization of surface functionalization, 2D layer materials have the potential to revolutionize targeted therapy and combination therapy. Photothermal therapy, immunotherapy, and chemotherapy have all shown improved efficacy when combined with 2D layer materials. Therefore, 2D materials are suitable for the capture and detection of serum biomarkers, imaging developers, energy conversion therapy, and kinetic therapy. Despite the challenges related to material stability, easy synthesis and biosafety, the future of 2D layered materials in cancer treatment remains bright after overcoming the flaws of the design itself. 2D materials have a broad future in the field of cancer diagnosis and treatment, which requires researchers to develop new materials in a targeted manner, while not forgetting clinical difficulties.
